# The New Mission of the American Medical Association, &c.

**Published:** 1865-05

**Authors:** 


### EDITORIAL AND MISCELLANEOUS.

### THE NEW MISSION OF THE AMERICAN MEDICAL ASSOCIATION.

The *Medical and Surgical Reporter*, of Philadelphia, a few months since, called attention to the losses of some of the physicians of Chambersburg, Pa., by the rebel "raid" on that place last summer, and proposed that the profession contribute to reimburse the same. In an editorial in the issue of April 15th the subject is again brought forward, amplified, and accompanied with a more explicit proposition to raise a fund for their relief. Nor does the *Reporter* now propose that the Chambersburg sufferers shall be the only recipients of this charity, but that it shall be extended "to all of our profession in the border States or elsewhere, who have been subjected to material losses by the ravages of the war." But we can only do the article justice by quoting from it at length:

"In view of the present aspect of affairs as connected with the rebellion, and the reasonable hope that our national troubles, so far as war and its ravages are concerned, are virtually ended, it is a proper time to propose an extension of the idea embraced in the suggestion in relation to the Chambersburg sufferers, to all of our profession in the border States or elsewhere, who have been subjected to material losses by the ravages of the war. It would be a noble instance of fraternal good will, if the medical profession of the United States were thus to unite in subscribing to a fund to make good to their brethren who have felt the ravages of the war, their office losses. The amount needed would not probably be very large, while the good done would be almost incalculable.

Within the next few weeks, many of our county and some of our State medical societies, and the American Medical Association, will meet. Let this subject be discussed, with a view to taking steps to do something practical and worthy of the medical profession of this great country. The plans might be systematized at the meeting of the American Medical Association, at Boston, in June, at which time a committee might be appointed to make an appeal to the profession of the whole country for funds. A portion of this committee might be named as an executive committee, to distribute the proceeds to those who need it. In this way it ought not to be difficult to raise a fund of fifty to a hundred thousand dollars for this purpose.

The American Medical Association could scarcely give greater evidence of its nationality and usefulness, just at this juncture, than by pursuing the course suggested. Indeed, if it gave up the greater part of its coming session to the work of perfecting a plan for raising and distributing such a fund as is proposed above, it would, in our view, be time well spent, and add greatly to the future influence and usefulness of the Association.

It is desirable to make the medical profession of this country, as far as possible, a unit in feeling, in purpose, and in action. This can best be done by the cultivation of intelligence and kindly feeling through our national medical organization. The war of the rebellion—now apparently, happily, near its close—has interfered somewhat with our intellectual and social progress, but if we are wise enough to avail ourselves of the opportunities offered us of re-establishing good feeling, it will give us the means of securing the intellectual advancement of our profession by securing their good will and co-operation. Many of our medical brethren have been deprived, by the ravages of the war, of their means of intellectual advancement The American Medical Association, by using its influence and its organization so as to aid them in replenishing their libraries and refitting their offices, would only be fulfilling its legitimate functions—in rather an unusual way to be sure, but then the occasion is an unusual one.

We invite correspondence, both on the specific subject of the Chambersburg sufferers, and on the general one of *all* those of our profession who have met with office losses by the ravages of the war, with a view of having something practical done for their benefit. *Let us help our brethren bear their burdens.*"

We certainly can hardly be wrong in supposing that the physicians of Chambersburg were unconscious of this attempt to thrust them as needy objects of commiseration upon the benevolent sensibilities of their professional brethren. "Ye have the poor always with you," is a revealed truth, that is every day attested by observation. We have forgotton who—but most likely it was some able divine—has divided this genus of persons into three species, as follows: the Lord's poor, the devil's poor, and poor devils. Now, if these members of a learned, elevating and honorable calling knew that they were to be thus publicly presented in the attitude of professional paupers, without presenting a protest against it, earnest and manly enough to have stifled such a movement, then have we no doubt among which division of the poor they are included. But we cannot do them the injustice of entertaining such a thought for a moment. The idea that the American Medical Association should lay aside its intellectual character and scientific labors, and "*give up the greater part of its coming session to the work of perfecting a plan for raising and distributing such a fund*," is as ludicrous as can well be conceived. The Knight of La Mancha, in his wildest moments, never indulged a more ridiculous fancy. The rebels, too, we suppose, are to be included in this Catholic scheme, for we are told it is to be a means of "re-establishing good feeling." This would be a fitting sequel to the efforts made at the last meeting of the Association, to furnish the rebels with what they needed more than anything else, namely: medical supplies. Had this sapient plan been carried out, its effect would have been to have returned a larger per centage of the rebels from their hospitals to the ranks, and in a shorter time, and had they accompanied this with a more rigorous starvation of our soldiers, prisoners in their hands, the war might possibly have been protracted another year, and thus offered us a longer opportunity of doing good to our enemies. But, should this project be consummated, and "fifty to one hundred thousand dollars" be obtained, we do not hesitate, in the name of the profession, to warn the almoners that the Association may appoint to dispense this bounty, not to show themselves in the Northwest in search of their objects of pity. We are poor enough out here, but not so "poor in spirit" as to receive such a proposition without feeling affronted. A people with manliness enough to take *arms* to repel a "raid" upon their soil, would not accept *alms* to cover incidental losses; while if they lack this manliness they do not deserve either sympathy or assistance in their sufferings.

---

[Interesting extract from a private letter from the Surgeon in charge of the Small Pox Hospital, Natchez, Miss.—Eds.

Mrs. ——, aged 26, was admitted to Hospital January 26, suffering from small pox of confluent form. Nothing remarkable took place until Feb. 3d, when she complained of severe pain in back and abdomen. Anodynes were given, but without benefit. 10 A. M.—She gave birth to a male child of about eight months, which survived four hours. The whole surface of the child was thickly covered at birth with pustules, some fully matured and breaking down and others partially filled. The mother continued to do well until the 7th, 8 P. M., when she was again delivered of a male child, apparently of about four or four and a half months, uterine life. This child was also thickly covered with pustules, but they were not so well filled as the first.

This was certainly a case of super fœtation; the difference in size and development of each child showing plainly that she conceived at different periods. There were two placentas. The woman sank from exhaustion, and expired at 4 A. M. Feb. 9th. The children were not preserved, for want of alcohol.

*Query 1st.*—If a fœtus suffers proportionately with the mother, would not vaccinating the mother afford immunity to the child in after life?

*Query 2d.*—Can it be possible for a woman to be pregnant, say four months advanced, and conceive again, with an uterus of a single cavity?

Eight hours after death. Post mortem in the above case revealed but one cavity, and nothing remarkable in the appearance of the uterus, excepting the mucous surface was thickly covered with pustules, none of which were well filled with pus.

---

U. S. A. Gen'l Hosp'l, Little Rock, Ark., Feb. 28, '65.
*Editors Chicago Medical Journal:*—

Gents:—Although a stranger to you, permit me to forward a very interesting case which came under my care, whilst on duty as Post Surgeon, at the "Mouth of White River," Ark. Private Cyrus Jones, of a colored regiment, was brought to the Hospital, Nov. 3d, 1864, suffering from an attack of "variola confluentia." At that time there was no medical officer assigned to duty at the Post. The patient was treated by the usual remedies, and appeared, as far as I can learn, to do well until about the 14th day after admission, when he complained of severe pain in his left foot. His comrades applied poultices of lins. pulv. and bread, but to no avail. His foot continued to grow worse until about Jan. 26th, at which time I was assigned to duty at that Post.

I found it had sloughed completely off at the tarsus; there was not a particle of hemorrhage, and the stump looked healthy. Patient's right leg at junction of lower with middle third, has also sloughed to such an extent as to expose the tibia for some four or five inches. The patient has recovered from the small pox and his general condition is very good, strange as it may appear. From the condition of his other foot, which was sloughing between the toes, and presents all the appearances of mortification, Dr. Mendenhall, Surgeon of the gunboat Tyler, and myself concluded to amputate it, which was performed at the anterior tuberosity of the tibia. He is now doing well and the stumps both look healthy.

I thought this case might be of interest to the profession, and forward for your favorable consideration what particulars I could learn regarding it previous to my being assigned to duty at this Post. There having been no medical officer stationed here the sick suffered very much and were much neglected.

H. S. HANNEN, M. D.,
Surg. in charge Col'd Ward.

---

*Death of Dr. Valentine Mott.*—The profession of medicine is suddenly called to lament the death of one of its most distinguished members, Dr. Valentine Mott. No name is so extensively known or so thoroughly identified with American Surgery as that of Dr. Mott. His long and eminently successful career, not only placed him in the very first rank of Surgeons in this country, but he stood as peer with the most renowned Surgeons of Europe. His reputation had won for him the highest honors accorded to the learned by the Scientific Societies of all countries, while at home he was regarded with veneration and affectionate esteem.

Dr. Mott was born at Glen Cove, Long Island, August 20, 1735, and was at the time of his death in his eightieth year. He was graduated in medicine at Columbia College, in 1806; soon after which he went to England and Scotland, where he continued his studies for several years.

He was long engaged as teacher in the several medical institutions of this city, and held at various times a Professorship in the Medical Department of Columbia College, in Rutgers' Medical College, in the College of Physicians and Surgeons, and in the University of New York.

Dr. Mott has not been a voluminous writer. Besides a number of reports and cases published in medical journals, his principal works were: "Travels in Europe and the East;" "Notes to a Translation of Velpeau's Operative Surgery:" "Anniversary Discourse before the New York Academy of Medicine," and "Clinical Reports."

He died, after a few days illness, ripe in years and full of honors.—*The New York Medical Journal.*

*Died.*—At Kinston, N. C., April 9th, 1865, Edward E. Lynn, M. D., Ass't Surg. 65th Illinois Infantry.

*Died.*—In this city, 16th ultimo, David Rutter, M. D., formerly a resident of Philadelphia.

*Married.*—In Lawrence, Kansas, April 4th, 1865, J. L. Prentiss, M. D., and Miss R. Anderson, both of Lawrence.

